# The Effect of Orally Administered Δ9-Tetrahydrocannabinol (THC) and Cannabidiol (CBD) on Obesity Parameters in Mice

**DOI:** 10.3390/ijms241813797

**Published:** 2023-09-07

**Authors:** Adi Eitan, Ofer Gover, Liron Sulimani, David Meiri, Betty Schwartz

**Affiliations:** 1 The Institute of Biochemistry, Food Science and Nutrition, The Robert H. Smith Faculty of Agriculture, Food and Environment, The Hebrew University of Jerusalem, Rehovot 9190401, Israel; adi.levy@mail.huji.ac.il (A.E.); ofer.gover@mail.huji.ac.il (O.G.); 2 Cannasoul Analytics, 9 Tarshish Industrial Park, Caesarea 3079822, Israel; liron@cannasoul.co.il; 3 The Laboratory of Cancer Biology and Cannabinoid Research, Department of Biology, Technion-Israel Institute of Technology, Haifa 32000, Israel; dmeiri@technion.ac.il

**Keywords:** obesity, HFD, endocannabinoids, THC, CBD

## Abstract

Prolonged cannabis users show a lower prevalence of obesity and associated comorbidities. In rodent models, Δ9-tetrahydrocannabinol (THC) and cannabidiol (CBD) from the plant *Cannabis sativa* L. have shown anti-obesity properties, suggesting a link between the endocannabinoid system (ECS) and obesity. However, the oral administration route has rarely been studied in this context. The aim of this study was to investigate the effect of prolonged oral administration of pure THC and CBD on obesity-related parameters and peripheral endocannabinoids. C57BL/6 male mice were fed with either a high-fat or standard diet and then received oral treatment in ramping doses, namely 10 mg/kg of THC or CBD for 5 weeks followed by 30 mg/kg for an additional 5 weeks. Mice treated with THC had attenuated weight gain and improved glucose tolerance, followed by improvement in steatosis markers and decreased hypertrophic cells in adipose epididymal tissue. Mice treated with CBD had improved glucose tolerance and increased markers of lipid metabolism in adipose and liver tissues, but in contrast to THC, CBD had no effect on weight gain and steatosis markers. CBD exclusively decreased the level of the endocannabinoid 2-arachidonoylglycerol in the liver. These data suggest that the prolonged oral consumption of THC, but not of CBD, ameliorates diet-induced obesity and metabolic parameters, possibly through a mechanism of adipose tissue adaptation.

## 1. Introduction

Obesity results from a long-term energy imbalance between excess calorie consumption and energy expenditure [1]. This leads to lipogenesis stimulation and excess fat storage as triglycerides in adipose tissue, resulting in the expansion of white adipose tissue and adipocyte hypertrophy, low-grade systemic inflammation, and changes in adipokine secretion, such as leptin and adiponectin [2]. This in turn leads to obesity-associated metabolic pathologies such as glucose intolerance, insulin resistance, and fatty liver disease [2].

The endocannabinoid system (ECS) plays a vital role in regulating appetite, metabolic processes, and energy balance both centrally and peripherally [3]. ECS is composed of cannabinoid receptor type 1 (CB1) and cannabinoid receptor type 2 (CB2), endogenous ligands, i.e., endocannabinoids, and the enzymes that synthesize and degrade them [3]. The endocannabinoids are bioactive lipids, the two most studied of which are N-arachidonoylethanolamide (AEA) and 2-arachidonoylglycerol (2-AG) [3]. In recent years, additional receptors, enzymes, and “endocannabinoid-like” mediators have been identified as part of the extended ECS. Among them, the congeners of AEA [N-acylethanolamines (NAEs)] and 2-AG [2-monoacyleglycerols (2-MAGs)] and N-acyl-amino acids (NAAAs) [4].

It is well established that obesity is associated with the dysregulation of the ECS, resulting in high endocannabinoid “tone”, which in turn leads to increased appetite, lipogenesis, adipogenesis, and a decrease in energy expenditure, which further exacerbates adiposity [5]. Consequently, the blockade of CB1 has been shown to exert appetite-suppressing anti-obesity properties and improve several pathological features associated with obesity, thus making it a potential therapeutic target for obesity treatment [6,7].

*Cannabis sativa* is the source of a unique set of compounds known as phytocannabinoids, from which the most abundant are Δ9-tetrahydrocannabinol (THC), the main psychotropic constituent of cannabis, and cannabidiol (CBD). Cannabis use has long been associated with increased appetite and is commonly used as an appetite stimulator in pathological conditions such as cancer and HIV [8]. Paradoxically, several epidemiological studies have established the association between cannabis exposure and reduced risk of obesity and metabolic diseases [9,10,11,12]. Moreover, cannabis extracts, as well as THC and CBD, have been shown to exert anti-obesity properties in animal models [13,14,15].

Most studies on cannabinoids in preclinical models to date employed injection as the mode of cannabinoid administration (i.e., intraperitoneal, subcutaneous, and intravenous). A limited number of studies have investigated the oral (per os) administration route. Absorption, bioavailability, metabolism, and overall effects are all influenced by the route of administration [16]. For example, in first-pass metabolites of THC, the psycoactive11-hydroxy-Δ9-tetrahydrocannabinol (11-OH-THC) is altered via the route of administration and could lead to biological relevant differences [17]. Moreover, the oral administration of cannabis products was demonstrated to produce prolonged effects compared with other administration routes [16]. Furthermore, cannabinoid receptors are distributed throughout the gastrointestinal tract, and some cannabinoid effects may be mediated peripherally [18]. Therefore, in the present study, we compared the anti-obesity properties of THC and CBD administrated per os and their obesity-related metabolic effects in a high-fat diet (HFD) mouse model.

## 2. Results

### 2.1. Pharmacokinetic Profile of Orally Administered THC and CBD

**Figure 1:**
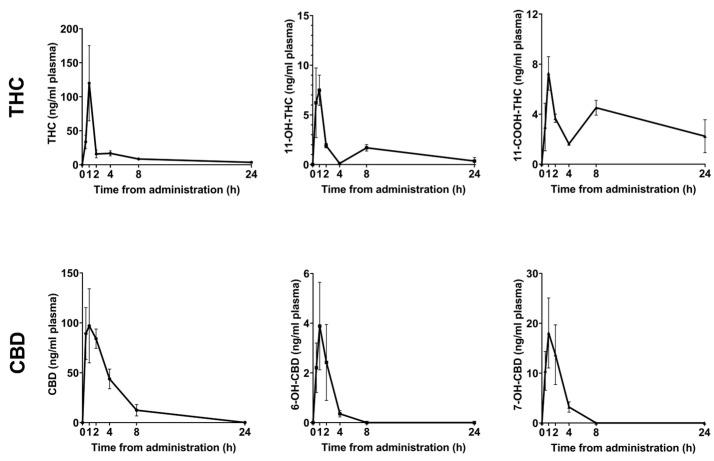
Pharmacokinetics profiles of THC, CBD, and their respective metabolites. Plasma concentration of THC and CBD and their respective first-pass metabolites, after oral administration of THC and CBD (30 mg/kg); *n* = 4 per time point per drug. Time points are presented as mean ± SEM.

To assess the availability of THC and CBD administrated per os, we first evaluated the pharmacokinetic profile of the oral administration delivery method of a single dose of 30 mg/kg. PK profiles of THC, CBD, and their respective metabolites are illustrated in Figure 1, and the key PK parameters of maximum concentration (Cmax), the time it takes to reach it (Tmax), and total exposure over time (area under the curve) are reported in Table 1. Notably, the plasma concentration of CBD was detected at 8 h but not at 24 h, and its metabolites were undetectable at 8 h, while plasma concentrations of THC and 11-COOH-THC were still detectable at 24 h. The metabolic effect of per os administration was evaluated over a period of 24 h using indirect calorimetry (Supplemental Figure S1A–C). Mice treated orally with THC had a trend of reduced locomotion and food intake and significantly reduced total energy balance relative to mice treated with vehicle or CBD.

### 2.2. The Effect of THC and CBD on Weight Gain, Caloric Intake, and Glucose Tolerance

**Figure 2:**
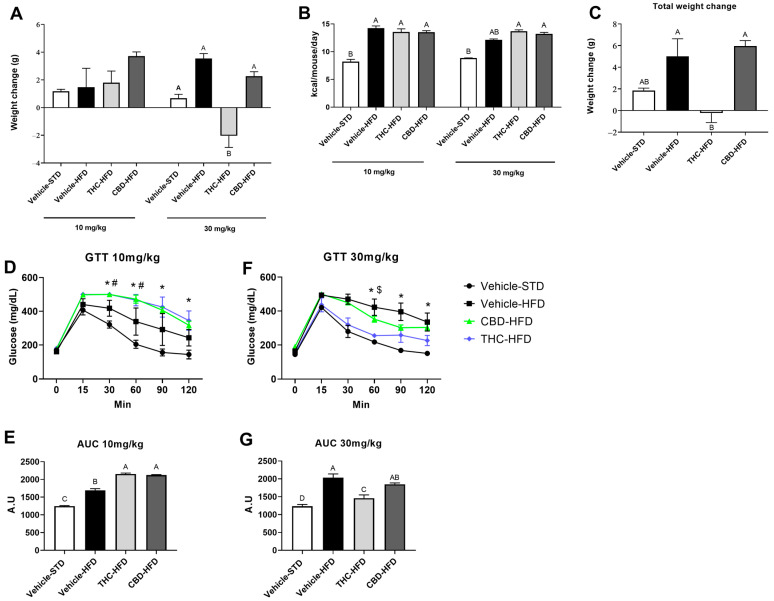
Effect of THC and CBD on weight gain, caloric intake, and glucose tolerance. Weight and food consumption were measured twice a week during the course of the treatment regime: (**A**) body weight change of HFD-fed mice after 5-week treatment with 10 mg/kg or 30 mg/kg of THC, CBD, or vehicle; (**B**) average caloric intake in HFD-fed mice treated with 10 mg/kg or 30 mg/kg of THC, CBD, or vehicle; (**C**) total weight change of the treatment regime (*n* = 5). A glucose tolerance test (GTT) was performed at the end of each dose treatment. Blood glucose concentration time course curves and area under the curve (AUC) were measured at the end of the 10 mg/kg treatment procedure (**D**,**E**) and at the end of the 30 mg/kg treatment procedure with THC, CBD, or vehicle (**F**,**G**), respectively (*n* = 3). Data and bar plots are shown as mean ± SEM. Different subscribed letters indicate significant differences between groups, *p* < 0.05. * *p* < 0.05 CBD vs. vehicle STD, # THC vs. vehicle STD, $ *p* < 0.05 CBD vs. THC. Statistical significance was determined via two-way ANOVA followed by Tukey’s post hoc test.

We evaluated the effect of prolonged oral consumption of THC and CBD in a mouse model of diet-induced obesity. In our treatment regime, we incorporated a ramping-dose method of THC and CBD, from 10 mg/kg to 30 mg/kg to account for the induction of tolerance, which is known to occur rapidly with prolonged cannabis use [19]. At 10 mg/kg, there were no significant changes between mice treated with THC or CBD compared with vehicle (Figure 2A). However, at 30 mg/kg, mice treated with THC had a significant decrease in body weight gain compared with mice treated with CBD (Figure 2A). Overall, THC inhibited weight gain on HFD-induced body-weight gain (final weight 38 ± 1.94), while vehicle and CBD-treated mice continued to gain weight (final weight 40 ± 1.97 and 45 ± 1.94, respectively) (Figure 2C). To determine whether this effect was exclusive to obese mice fed HFD, we conducted the same treatment regime in mice fed standard diet (STD). Treatment with THC or CBD did not significantly affect weight gain at either dose (Supplemental Figure S2). In each diet regime, irrespective of treatment, the average daily energy intake per mouse at the respective diet appeared to be similar over the study period (Figure 2B, Supplemental Figure S2B).

To assess the effect of THC or CBD administration on the regulation of blood sugar levels, a glucose tolerance test was performed at the end of each treatment regime. Mice fed HFD developed glucose intolerance irrespective of the treatment at a dose of 10 mg/kg, although a significant increase in glucose levels was observed in THC- and CBD-treated mice, compared with the vehicle-treated HFD mice (Figure 2D,E). At the dose of 30 mg/kg, THC reversed this effect and showed significant improvement in glucose tolerance (Figure 2F,G).

### 2.3. The Effect of THC and CBD on the ECS in Liver and Adipose Tissues

As obesity is characterized by the dysregulation of the endocannabinoid tone [20], we next investigated the effect of THC and CBD treatment on the prevalence of different components of the ECS. The levels of the well-known endocannabinoids AEA and 2-AG were determined in epididymal fat and liver tissue via LC-HRMS, as well as other members of NAE and 2-MAG families, and members from the family of NAAAs. In the epididymal tissue, the levels of AEA and 2-AG were comparable between all treatment groups; however, obese mice demonstrated decreased levels of some NAEs (DHEA, α-LEA, LEA, and PEA) compared with lean mice, although the last two were not significantly different when compared to CBD-treated mice (Table 2). In contrast, 2-LG levels were increased in obese mice compared with lean mice, although this reached significance only in mice treated with vehicle or CBD. In the liver tissue, AEA levels were similar between all treatment groups; however, all obese mice, regardless of treatment, demonstrated decreased levels of NAEs (LEA, PEA, and SEA) and NAAAs (NDH-Gly, NL-Gly, and NP-Gly) compared with lean mice (Table 2). CBD treatment further decreased the levels of DHEA, NA-Gly, and NA-Ser compared with vehicle-treated STD mice. Mice treated with THC or CBD demonstrated decreased levels of 2-AG compared with vehicle-treated mice fed both HFD and STD, although only CBD treatment reached significance.

**Figure 3:**
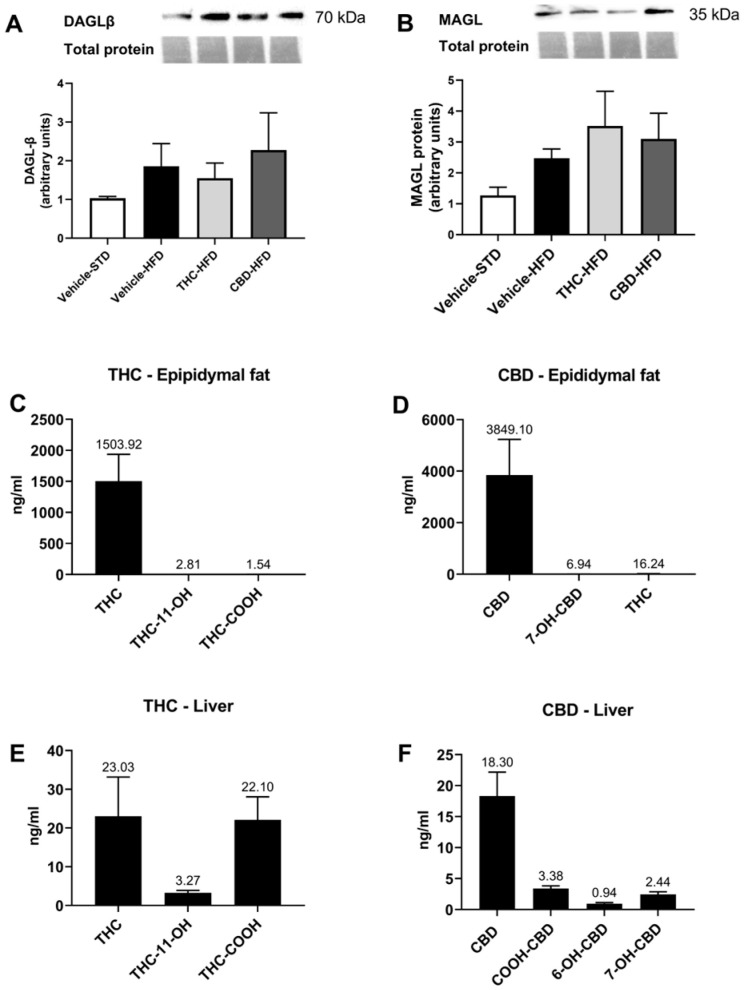
Expression of DAGL-β, MAGL, and phytocannabinoid metabolites in liver and adipose tissue. Protein levels of key enzymes involved in the synthesis and hydrolysis of 2-AG, DAGL-β (**A**), and MAGL (**B**), respectively (*n* = 2–4). Levels of THC, CBD, and their metabolites were assessed at the end of the treatment regime, as described in the Materials and Methods section, in epididymal fat (**C**,**D**) and liver tissue (**E**,**F**) (*n* = 4). Data are shown as mean ± SEM.

We then examined whether the attenuation of 2-AG was due to the altered expression of DAGL-β and/or MAGL, the main enzymes responsible for its synthesis and degradation, respectively. No changes were detected in protein levels of the two enzymes, indicating that the changes in 2-AG levels observed in the liver were not the result of decreased DAGL-β or increased MAGL expression (Figure 3A,B). 

Furthermore, to confirm that the examined tissues were exposed to pharmacological levels of THC and CBD, we analyzed the liver and adipose tissues for the presence of these phytocannabinoids and their respective metabolites in the THC- and CBD-treated groups (Figure 3C–F). As expected, high concentrations of THC and CBD with low levels of metabolites were revealed in the epididymal fat in the respective treatment groups (Figure 3C,D). CBD-treated mice also showed traces of THC in epididymal tissue, although this might be due to technical issues. Nonetheless, we analyzed CBD extract to verify that there were no traces of THC (Supplementary Figure S3).

### 2.4. The Effect of THC and CBD on Diet-Induced Steatosis

**Figure 4:**
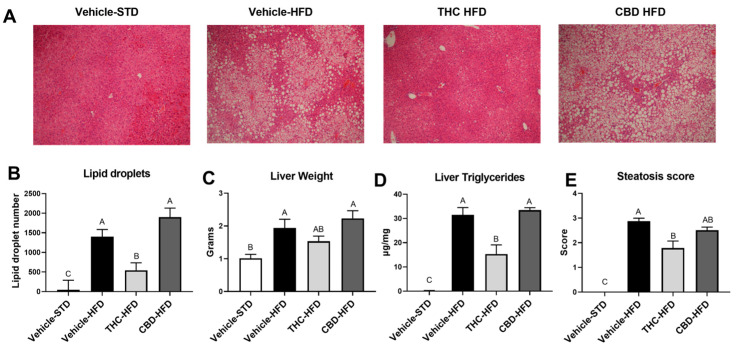
Effect of THC and CBD on obesity-induced steatosis. Obesity-induced steatosis was assessed at the end of the treatment regime in mice treated with CBD or THC: (**A**) representative liver tissue sections stained with H&E (magnification × 100); (**B**) morphological analysis of liver droplet density (*n* = 5); (**C**) weights of liver; (**D**) levels of hepatic triglycerides (*n* = 3); (**E**) the severity of steatosis was blindly scored as described by Liang et al. [21] (*n* = 3). Data are shown as mean ± SEM. Statistical significance was determined via one-way ANOVA followed by Tukey’s post hoc test. Different subscribed letters indicate significant differences between groups, *p* < 0.05.

A key component of obesity-associated metabolic dysregulation is hepatic steatosis. Therefore, we tested for the manifestation of enlarged hepatic vacuoles and increased lipid droplets. THC, but not CBD, reduced the abundance of hepatic lipid droplets (Figure 4A,B). In addition, THC treatment showed a trend toward a reduction in enlarged liver, less elevation of hepatic triglyceride content, and reduced steatosis score, indicating an overall minimal accumulation of excess fat (Figure 4C–E). 

### 2.5. The Effect of THC and CBD on Adiposity and Inflammation

**Figure 5:**
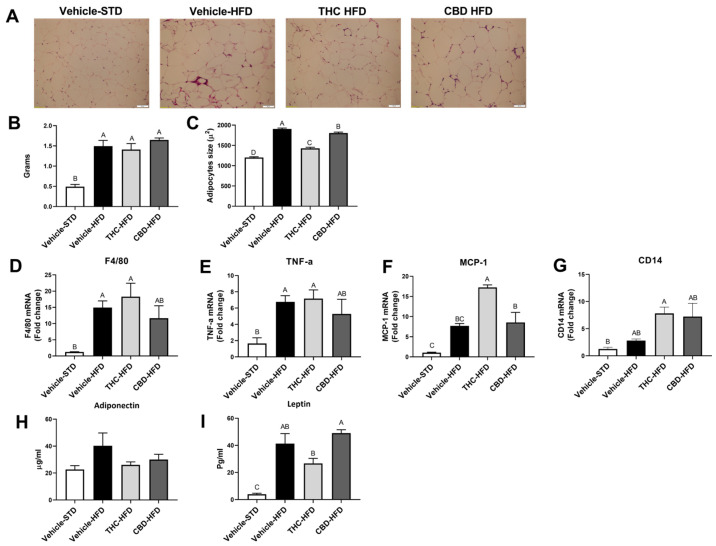
Effect of THC and CBD on adiposity, adipocytes, and inflammation markers. Epididymal fat was removed from mice and analyzed after the treatment regime with THC, CBD, or vehicle treatment: (**A**) representative adipose tissue sections stained with H&E (magnification × 200); (**B**) weight of fat pads; (**C**) quantitative analysis of adipocyte size in epididymal tissue (*n* = 5); (**D**–**G**) mRNA levels of inflammatory genes in epididymal adipose tissue (*n* = 4). Serum levels of adiponectin levels (**H**) and leptin (**I**) (*n* = 3). Data are shown as mean ± SEM. Statistical significance was determined via one-way ANOVA followed by Tukey’s post hoc test. Different subscribed letters indicate significant differences between groups, *p* < 0.05.

Adipose tissue dysfunction, specifically that of visceral fat, plays a major role in glucose intolerance in diet-induced obese mice [22]. Thus, we determined if the improved glucose tolerance in CBD- and THC-treated mice was due to lowering adiposity, inflammation, or adipokine secretion. Treatment with THC and CBD had no effect on fat pad weights compared to vehicle-treated HFD mice (Figure 5B). However, THC-treated HFD mice had significant shrinkage in adipocyte size compared with vehicle-treated HFD mice and presented smaller adipocytes also compared to the CBD-treated mice (Figure 5A,C).

In terms of inflammation, HFD-fed mice presented elevated expression of F4/80 and TNF-α inflammatory markers compared to vehicle-STD, as expected (Figure 5D,E). Moreover, THC significantly increased the expression of both the chemokine MCP-1 compared to vehicle-HFD and CBD, and of CD14 compared to vehicle-STD (Figure 5F,G), an indicator of inflammation. Measurement of adiponectin revealed no changes between the experimental groups (Figure 5H). However, treatment with THC significantly reduced the levels of leptin compared to CBD treatment (Figure 5I).

### 2.6. The Effect of THC and CBD on Lipid Metabolism Gene Expression

**Figure 6:**
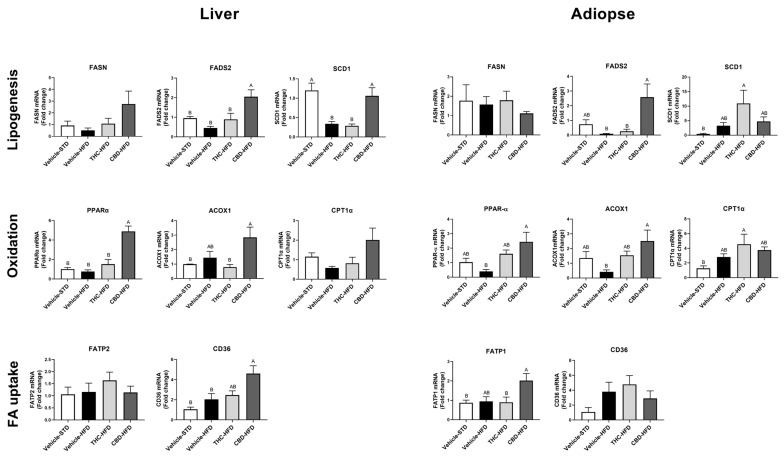
The effect of THC and CBD on liver and adipose tissue mRNA expression of genes involved in lipid metabolism. Expression profile of genes involved in lipogenesis (*FASN*, *FADS2*, and *SCD1*), fatty acid uptake (*FATP2*, *FATP1*, and *CD36*), and lipid oxidation (*PPARα*, *ACOX1*, and *CPT1*α) (*n* = 4–5). *TBP* and *PPIA* were used as housekeeping genes for liver and adipose tissue, respectively. The results were calculated as ΔΔCt and normalized to data from vehicle STD. Data are shown as average ± SEM. Statistical significance was determined using one-way ANOVA followed by Tukey’s post hoc test. Different subscribed letters indicate significant differences between groups, *p* < 0.05.

To assess whether the effects of THC and CBD observed in the liver and adipose tissue were correlated to changes in the gene expression of lipid metabolism, we examined mRNA levels of lipogenic and lipid oxidation markers. In the liver, treatment with CBD increased the transcription of the typical lipogenic genes *FADS2* and *SCD-1*, compared with THC- and vehicle-treated HFD mice (Figure 6). Furthermore, CBD treatment increased the expression of the lipid oxidation markers *ACOX1* and *PPAR-α* compared with THC treatment, while the latter was also significantly increased compared with vehicle HFD (Figure 6). In addition, CBD increased transcription of fatty acid transporter *CD36* compared to vehicle-treated groups. A similar trend was shown in adipose tissue, in which CBD increased the transcription of the lipogenic gene *FADS2*, in addition to the oxidative genes, *PPAR-α* and *ACOX1*, and fatty acid transporter *FATP1* (Figure 6).

## 3. Discussion

Previous studies have investigated the anti-obesity properties of treatment with single cannabinoids or cannabis extracts [13,14,15]. However, to our knowledge, we are the first to incorporate a noninvasive oral route of administration method to compare the prolonged treatment of THC and CBD in an animal model of obesity. In this study, we demonstrated that per os treatment with THC, but not CBD, prevented HFD-induced body weight gain and improved metabolic alterations after diet-induced obesity in C57BL/6 male mice. Because tolerance is known to occur rapidly during cannabis use [19], we examined the effect of increased dose during the treatment regime. We observed a contrasting effect of THC on weight gain and glucose tolerance in the two doses employed in this study. 

We selected the oral route administration since it represents a comparable form to human medical cannabis consumption. Moreover, it has been suggested that intraperitoneal injections, the route most frequently used for cannabis extracts in animal models, may produce biological effects that stem from the accumulation of active cannabinoids such as THC in the brain and the activation of the CB1 receptor in a manner that is distinct from that which occurs in typical cannabis use [17]. Our results show that the oral administration of THC and CBD at the concentration implemented in our experiment produced plasma concentrations comparable to the range seen in other administration routes in mouse models [16,17]. 

Several studies have provided evidence that the ECS plays an important role in the development of obesity [3,20]. Obesity is characterized by an increased endocannabinoid tone, which in turn leads to metabolic disorders that contribute to weight gain, lipogenesis, insulin resistance, and dyslipidemia [5,20]. By contrast, decreased CB1 activity in an obese setting is linked to weight reduction and overall improved metabolic state [6,7]. Though THC shares the ability of AEA and 2-AG to activate both CB1 and CB2 receptors [23], epidemiologic studies have associated cannabis use with lower body mass index (BMI) and lower prevalence of obesity-associated metabolic diseases such as nonalcoholic fatty liver disease (NAFLD) and diabetes compared with non-users [9,10,11,12].

In our study, CBD treatment exerted no effect on weight gain. However, THC demonstrated a biphasic effect, in which 10 mg/kg of THC increased weight gain, similarly to that of HFD-fed mice, while 30 mg/kg of THC decreased weight gain. This biphasic effect was not correlated with food intake, which was unaffected throughout the experiment. In agreement with these results, a previous report demonstrated that a reduction in food intake is not sufficient to produce prolonged anti-obesity effects via CB1-mediated pathways [6].

The contrasting effect of THC on weight gain could be associated with its ability to produce greater tolerance when administered in a ramping-dose procedure [24]. Studies have shown that THC tolerance, caused by prolonged cannabis use, leads to the loss and desensitization of CB1; thus, the associated phenotype could reflect a decrease rather than an increase in CB1 activity [25]. Supporting this notion, an opposed effect was also observed on glucose tolerance in mice treated with THC. 

In line with this, previous studies demonstrated the induction of glucose intolerance by endocannabinoid agonists and an improvement in insulin sensitivity by CB1 antagonists [26,27]. Our findings indicated an elevation in inflammatory markers in adipose tissue in mice treated with CBD and THC, suggesting that the improvement in glucose tolerance using THC is not related to reduced inflammation [28].

One of our aims in this study was to evaluate the modulatory effect of THC and CBD on the peripheral ECS, specifically in visceral adipose tissue and liver. We did so by examining the levels of the main endocannabinoids AEA and 2-AG, as well as other family members of NAEs, 2-MAGs, and NAAAs that together act as mediators within the endocannabinoidome [4]. Although THC and CBD substantially accumulated in target tissues, obese mice demonstrated decreased levels of AEA and 2-AG congeners, mainly NAEs and NAAAs, compared with lean mice. This could be the result of the fatty acid composition of the HFD used in this study, rather than the presence of the high-fat intake itself, as previously suggested [29,30].

Based on the fatty acid compositions of the two diets used in our study (Supplementary Figure S1), we speculate that the inverse proportions of polyunsaturated fatty acids compared with saturated fatty acids and monounsaturated fatty acids, together with similar ω6:ω3 ratios in the HFD, affected the biosynthetic precursors of these lipids in a way that eventually led to decreased levels in the tissues examined from HFD-fed mice. In agreement, 2-AG and AEA levels in the epididymal fat were unaffected by diet or treatment. In contrast, the 2-LG derivate of 2-MAGs was increased in the epididymal tissue of HFD-fed mice treated with vehicle and CBD compared with lean mice. This observation could reflect the mechanism in which 2-MAGs are formed in the adipose tissue, during the mobilization of stored triglycerides (TGs) [31]. Indeed, the adipocyte cell size of mice from all treatment groups corresponded to the levels of 2-LG seen in the epididymal tissue. 

In the liver, mice treated with CBD exhibited decreased levels of 2-AG, suggesting specific modulation with CBD. The decreased levels of 2-AG cannot be explained by corresponding level changes in the main synthesizing enzyme of 2-AG in the liver, DAGLβ, nor by its hydrolyzing enzyme, MAGL, which were similar in all treatment groups. It is possible that the expression of the enzymes did not result in changes in enzyme activity, as seen previously [32]. Alternatively, it is possible that the low levels of 2-AG seen in the liver reflect alterations in the metabolism of the phytocannabinoid that occur via different biosynthetic enzymes [33]. 

Previous studies have shown that elevated levels of 2-AG, the main CB1 endogenous agonist, increase the transcription of lipogenic genes and increase TG production in the liver, thus contributing to the development of diet-induced fatty liver [34,35,36]. However, CBD treatment increased the transcription of lipogenic genes and of PPARα in the liver, and although the latter induces the expression of genes involved in FA oxidation, the upregulation is more likely due to the efflux of free fatty acids (FFA) to the liver [2]. Subsequently, CBD had no beneficial effect on liver steatosis. Conversely, however, THC treatment improved liver steatosis, and these changes were not associated with transcriptional changes of steatogenic genes. It is possible that THC reduced liver steatosis indirectly by limiting the influx of fatty acids originating from adipose tissue, as indicated by the significant decrease in adipocyte size compared with vehicle-treated HFD and CBD-treated groups. Indeed, large adipocytes from visceral fat undergo higher rates of lipolysis, thus increasing the circulation of FFA, consequently leading to the ectopic deposition of fats in the liver [2]. However, the analysis of gene expression in epididymal tissue revealed no significant effect of THC on gene-associated lipid metabolism regulation. 

THC exerts a broad systemic effect, demonstrated by the decreased energy balance seen in acute administration using indirect calorimetry (Supplementary Figure S2), which resulted in weight loss and reduction in plasma leptin compared with CBD, suggesting decreased fat mass and improved leptin sensitivity in the context of obesity [37,38]. Taken together, it is possible that the improved metabolic state induced by THC resulted from modulated energy metabolism, possibly mediated by leptin, and adipose adaptation. 

An important limitation of the study arises from the relatively small number of animals per group; thus, further research is recommended. Furthermore, the study was performed on male mice, so it would be beneficial to examine sex differences in oral THC treatment in an obese setting. 

## 4. Materials and Methods

### 4.1. Animals

All procedures were approved by the animal ethics committee of the Hebrew University of Jerusalem (approval number: AG-23-15437-3). Four-week-old C57BL/6 male mice were purchased from Envigo (Rehovot, Israel) and housed in polycarbonate cages under a 12 h light/dark cycle-controlled temperature and constant humidity. Mice were fed ad libitum with either a standard diet (STD; 2018sx; Harlan Teklad) containing 70% carbohydrates, 20% protein, and 10% fat, or a high-fat diet (HFD; TD.06414; Harlan Teklad) containing 60% fat, 20% protein, and 20% carbohydrates. The fatty acid profile of the STD and HFD are shown in Supplemental Table S1.

### 4.2. Treatments

Pure THC (BOL pharma, Revadim, Israel) or CBD (Tikun Olam Ltd., Tel-Aviv, Israel) were dissolved in olive oil. Mice were fed with HFD or STD for 14 weeks, and then randomly divided into treatment groups (*n* = 5). The dosing schedule was based on consideration of “frequent cannabis use” as determined elsewhere [39]. Mice were administered via noninvasive oral delivery using a micropipette of 10 µL of THC, CBD, or vehicle control (olive oil) at 10 mg/kg three times a week for five weeks, followed by an additional five weeks of 30 mg/kg treatment. At the end of the treatments, mice were euthanized via isoflurane overdose, and their blood was collected, and serum leptin and adiponectin levels were determined using a commercial ELISA kit (Ray Biotech, Peachtree Corners, GA, USA). Liver and adipose tissues were also collected and further analyzed.

### 4.3. Pharmacokinetics (PK)

For PK assessments, mice were deprived of food for 12 h and then orally administered 30 mg/kg of THC or CBD (*n* = 4 per time point). Then, 4 h after administration, mice were given food ad libitum for the remainder of the experiment. Mice were anesthetized with isoflurane at 0.5, 1, 2, 4, 8, and 24 h after administration, and blood was collected from the orbital sinus. Plasma was isolated via centrifugation at 3000 RPM at 4 °C for 15 min and transferred into polypropylene tubes, which were immediately frozen and stored at −80 °C for future analysis of THC and CBD metabolites.

### 4.4. Metabolic Cages 

Control and treated mice (*n* = 4) were housed individually in metabolic cages. The mice were acclimated in metabolic cages for two days and then fasted for 12 h before receiving treatment with either vehicle, THC, or CBD at a concentration of 30 mg/kg. Two hours after treatment, mice were given food ad libitum for the remaining 24 h. All measurements were obtained using an indirect calorimetry system (Promethion, Sable systems, Las Vegas, NV, USA). The volume of O_2_ and CO_2_ inspired or expired by each animal was recorded, and the relative respiration rate (RER) was calculated as the ratio of vCO_2_ to vO_2_. Data collected continuously included food consumption and locomotor activity. Data were exported using Expedata software (Ver 1.9.14, Sable) and converted using Macro interpreter (Sable) to CSV and XML files. Downstream analysis was carried out using CalR (ver 1.3) [40] and GraphPad Prism 9 software.

### 4.5. Glucose Tolerance Test

Intraperitoneal glucose tolerance tests were performed in mice following 6 h of fasting prior to the injection of the glucose solution (2 g/kg). Blood glucose from the tail ends was determined using a glucometer (FreeStyle Optimum Neo glucometer, Abbott, IL, USA); AUC between 0 and 120 min was determined using the GraphPad Prism 9 software.

### 4.6. Liver Triglyceride Quantification

Briefly, 80 mg of liver tissue was homogenized in 0.5 mL of ice-cold 1:1 methanol–Tris (50 mM Tris buffer pH 8) solution. The homogenate was washed twice with 1 mL ice-cold chloroform–methanol (1:1 *v*/*v*), and 0.5 mL of 50 mM Tris pH was added and vortexed, and then the mixture was centrifuged at 3000× *g* at 4 °C for 10 min. The organic phase was transferred to a glass tube and underwent two more extractions with ice-cold chloroform. Then, 1 mL of 5% Triton X-100 in chloroform was added and mixed using vortex. Chloroform was evaporated with N_2_ gas at 32 °C, and triglycerides were reconstituted with 1 mL of ddH_2_O. The extracted triglycerides were measured using a triglyceride colorimetric kit according to manufacturer instructions (Cayman Chemicals, Ann Arbor, MI, USA).

### 4.7. Histology and Microscopy

Adipose and liver tissues were embedded in paraffin and sectioned to 5 µM thickness on superfrost slides. Slides were stained using hematoxylin (Hebrew University pathology core facility). The stained slides were photographed using a Canon microscope with a mounted Olympus camera. Adipose cell size and hepatic lipid droplet counts were calculated using Fiji software (ver 1.53) [41].

### 4.8. Liquid Chromatography–High-Resolution Mass Spectrometry (LC-HRMS) Chemical Analysis

The analysis of endocannabinoids, endocannabinoid-like lipids, and pure THC and CBD from plasma, liver, and adipose tissue samples was performed using Thermo Scientific ultra HPLC system coupled with a Q ExactiveTM Focus Hybrid Quadrupole-Orbitrap MS, according to a method developed and validated by David Meiri and colleagues [42].

### 4.9. Western Blot

Liver tissue samples were homogenized in a RIPA buffer (Santa Cruz Biotechnology, Dallas, TX, USA) supplemented with a complete protease inhibitor cocktail (Sigma, ST. Louis, MO, USA) and centrifuged at 20,000× *g* for 15 min. The supernatant was collected, and protein concentration was measured with a BCA protein quantification kit (Pierce, Thermo Fisher, Waltham, MA, USA). Then, 60 µg of protein was loaded in each well and separated on 12% precast gels (Bio-Rad, Hercules, CA, USA) and blotted onto nitrocellulose membrane (Bio-Rad), which were blocked in 5% BSA and incubated with anti-DAGLβ (Cell Signaling, Danvers, MA, USA, Cat# D4P7C) dilution 1:1000 and anti-MAGL (Abcam, Cambridge, UK, Cat# ab24701) dilution 1:200. Blots were incubated with HRP-conjugated anti-rabbit secondary antibody (Abcam, ab6721) and detected using ECL solution (Cytiva, Amersham, UK). Imaging and quantification were performed with ChemiDoc (Bio-Rad).

### 4.10. RNA Extraction and Real-Time Quantitative PCR

RNA was extracted from the hepatic tissues using the NucleoSpin RNA extraction kit (Mecherey-Nagel, GmbH, Düren, Germany) according to the manufacturer’s instructions. Additionally, RNA was extracted from the adipose tissue using RNeasy Lipid Tissue Mini Kit (Qiagen, Hildan, Germany) according to manufacturer instructions. Briefly, 1 µg of total RNA was used to create cDNA using the qScript cDNA Synthesis Kit (Quantabio, Beverly, MA, USA). Relative gene expression was analyzed on QuantStudio 1 Real-Time PCR using SYBR^®^ Green-Based qPCR (Thermo Fisher). Real-time PCR primers are listed in Supplementary Table S2.

### 4.11. Statistics

Statistical analyses were performed using GraphPad Prism 9 (GraphPad software, version 9.0.0). Unless otherwise stated, data are expressed as mean ± SEM. Statistical differences were tested using one-way or two-way ANOVA, followed by Tukey’s post hoc test. Regarding the PK data analysis, Cmax and area under the curve (AUC) were measured using GraphPad Prism 9. The times maximum concentrations were reached (Tmax) were determined via visual inspection of averaged data. A difference of *p* ˂ 0.05 was considered statistically significant. 

## 5. Conclusions

In conclusion, the present findings provide evidence for the ability of THC to improve obesity-related metabolic complications when administered orally in ramping doses. The limited effect of CBD demonstrated in our study suggests that the low prevalence of obesity and metabolic diseases seen in cannabis users is mainly attributed to the presence of THC. Further studies are needed to explore the mechanism through which THC, via adipose adaptation and/or leptin sensitivity, reduces obesity-induced hepatic steatosis. 

## Figures and Tables

**Table 1:** PK parameters for THC, CBD, and their respective metabolites after acute oral administration (30 mg/kg).

		C_max_ (ng/mL)	AUC (ng·min/mL)	T_max_ (h)
THC	Δ^9^-THC	119 ± 55.3	295.2 ± 75.4	1
	11-OH-THC	7.4 ± 1.5	31.66 ± 8.4	1
	11-COOH-THC	7.25 ± 1.34	80.3 ± 23.3	1
CBD	CBD	96.9 ± 37.1	500 ± 115	1
	6-OH-CBD	3.88 ± 1.76	8.7 ± 4	1
	7-OH-CBD	18 ± 7.03	48.9 ± 16.44	1

PK parameters of THC and CBD in plasma after oral administration of THC or CBD (30 mg/kg). Data are shown as mean ± SEM with *n* = 4 animals per time point. THC, Δ9-tetrahydrocannabinol; 11-OH-THC, 11-hydroxy-Δ9-tetrahydrocannabinol; 11-COOH-THC, 11-Nor-9-carboxy-Δ9-tetrahydrocannabinol; CBD, cannabidiol; 6-OH-CBD, 6-hydroxy-cannabidiol; 7-OH-CBD, 7-hydroxy-cannabidiol.

**Table 2:** Levels of endocannabinoids in epididymal fat and liver.

	Lipid Family	ECs (ng/g Tissue)	Vehicle-STD	Vehicle-HFD	THC-HFD	CBD-HFD
Fat	NAEs	AEA	6.7 ± 0.2	8.5 ± 0.7	6.9 ± 0.2	8.1 ± 0.4
		α-LEA	0.9 ± 0.1 ^a^	0.2 ± 0.01 ^b^	0.2 ± 0.01 ^b^	0.2 ± 0.02 ^b^
		DHEA	2.6 ± 0.1 ^a^	1.8 ± 0.1 ^b^	1.6 ± 0.1 ^b^	1.7 ± 0.09 ^b^
		LEA	43.7 ± 1.5 ^a^	31.1 ± 3 ^b^	24.2 ± 1.8 ^b^	33.6± 3 ^ab^
		OEA	43.3 ± 4	54 ± 5.4	39.6 ± 3.5	55.7± 4.1
		PEA	54.4 ± 3.7 ^a^	36.8 ± 1.7 ^b^	30.4 ± 4.6 ^b^	41 ± 1.9 ^ab^
		SEA	112 ± 9.4	130 ± 11.5	115 ± 5.9	127 ± 2.1
		DEA	1.4 ± 0.7	1.2 ± 0.07	1.3 ± 0.04	1.5 ± 0.09
	2-MAGs	2-AG	144 ± 108	147 ± 32	104 ± 14	164 ± 45
		2-LG	352 ± 86 ^b^	2534 ± 601 ^a^	1128 ± 250 ^ab^	2773 ± 739 ^a^
	NAAAs	NL-Gly	2.8 ± 0.2	1.5 ± 0.3	1.3 ± 0.2	1.5
		NP-Gly	0.6 ± 0.1	1 ± 0.2	0.8 ± 0.1	0.9 ± 0.09
Liver	NAEs	AEA	28.6 ± 3.6	23.8 ± 4.8	20.6 ± 4.1	12.9 ± 0.5
		DHEA	8.8 ± 1 ^a^	6 ± 1.5 ^ab^	4.6 ± 0.6 ^ab^	2.9 ± 0.1 ^b^
		LEA	69.4 ± 10.7 ^a^	36.8 ± 7.8 ^b^	30.3 ± 4.5 ^b^	20.8 ± 1.5 ^b^
		OEA	40.5 ± 7.9	33.6 ± 2.4	24.5 ± 6.6	25.5 ± 1.8
		PEA	326 ± 27 ^a^	157 ± 16 ^b^	107 ± 4.7 ^b^	100 ± 8.6 ^b^
		SEA	288 ± 39 ^a^	134 ± 27 ^b^	111 ± 4.7 ^b^	75.2 ± 3.6 ^b^
	2-MAGs	2-AG	1975 ± 171 ^a^	1763 ± 572 ^a^	767 ± 102 ^ab^	390 ± 23 ^b^
		2-LG	12,682 ± 2214 ^a^	5748 ± 2132 ^b^	4112 ± 1208 ^b^	1951 ± 341 ^b^
	NAAAs	NL-Gly	359 ± 34 ^a^	49 ± 41 ^b^	95 ± 14 ^b^	50.3 ± 5.9 ^b^
		NP-Gly	695 ± 49 ^a^	366 ± 70 ^b^	284 ± 33 ^b^	177 ± 18 ^b^
		NA-Gly	228 ± 14 ^a^	117 ± 44 ^ab^	118 ± 28 ^ab^	61.9 ± 10.8 ^b^
		NDH-Gly	244 ± 15.9 ^a^	106 ± 33.3 ^b^	69.1 ± 13.3	45.3 ± 6.1
		NA-Ser	83.3 ± 4.5 ^a^	46.3 ± 19.9 ^ab^	34.5 ± 8 ^ab^	15 ± 1.9 ^b^
		NA-GABA	6.1 ± 1.4	6 ± 2.5	4.6 ± 1	1.5 ± 0.2
		NA-Ala	126 ± 12.1 ^a^	91.8 ± 32.7 ^b^	96.7 ± 21.3 ^b^	41.2 ± 5.5 ^b^

Data are shown as mean ± SEM with *n* = 4 animals per group. Statistical significance was determined using one-way ANOVA followed by Tukey’s post hoc test. Different subscribed letters indicate significant differences between groups, *p* < 0.05; 2-AG, 2-arachidonoyl glycerol; AEA, arachidonoyl ethanolamide; DHEA, docosahexanoyl ethanolamide; LEA, linoleoyl ethanolamide; OEA, oleoyl ethanolamide; PEA, palmitoyl ethanolamide; SEA, stearoyl ethanolamide; DEA, docosatetraenoyl ethanolamide; α-LEA, linolenoyl ethanolamide; 2-LG, linoleoyl glycerol; NL-Gly, N-linoleoyl glycine; NP-Gly, N-palmitoyl glycine; NA-Gly, N-arachidonoyl glycine; NDH-Gly, N-docosahexaenoyl glycine; NA-Ser, N-arachidonoyl serine; NA-GABA, N-arachidonoyl gamma-aminobutyric acid; NA-Ala, N-arachidonoyl alanine.

## Data Availability

The data underlying this article will be shared upon reasonable request by the corresponding author.

## Supplementary Materials

The following supporting information can be downloaded at: https://www.mdpi.com/article/10.3390/ijms241813797/s1.
