# Supplementary material for: The Effect of Orally Administered Δ9-Tetrahydrocannabinol (THC) and Cannabidiol (CBD) on Obesity Parameters in Mice
Source: Int J Mol Sci. 2023 Sep 7;24(18):13797. doi: 10.3390/ijms241813797 (PMC10530777; doi:10.3390/ijms241813797)
Supplement: Supplementary file 1 [file ijms-24-13797-s001.zip › ijms-2575132-supplementary.pdf]

**Supplementary Information**

# Effect of orally administered $\Delta^9$-Tetrahydrocannabinol (THC) and cannabidiol (CBD) on obesity parameters in mice

Adi Eitan, Ofer Gover, Liron Sulimani, David Meiri, and Betty Schwartz

**Table S1: Fatty acid composition of the STD and HFD used in this study**

| | HFD | STD |
|---|---|---|
| Total fat (g/kg) | 342.7 | 62 |
| Saturated fat (g/kg) | 119.2 | 0.63 |
| C14:0 myristic | 3.1 | - |
| C16:0 Palmitic | 74.6 | 0.49 |
| C18:0 Stearic | 41.5 | 0.14 |
| Monounsaturated fat (g/kg) | 134 | 9.1 |
| C16:1 Palmitoleic | 6.2 | - |
| C18:1 Oleic | 127.8 | 8.4 |
| Polyunsaturated fat (g/kg) | 77.2 | 23.8 |
| C18:2 Linoleic | 71.7 | 21.7 |
| C18:3 Linolenic | 5.5 | 0.21 |
| ω6:ω3 ration | 13 | 10 |

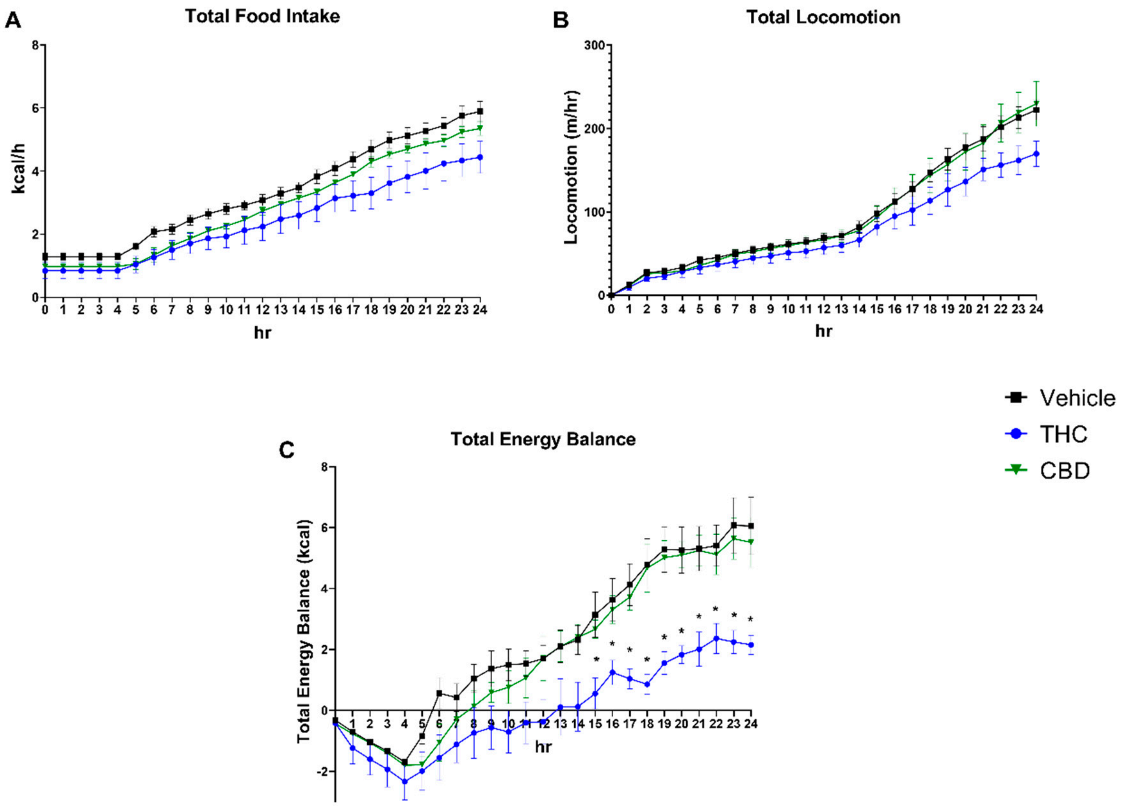


**Figure S1: Food intake, locomotion, and energy balance after single dose of 30 mg/kg of THC and CBD.** Mice were treated with vehicle, THC (30 mg/kg), or CBD (30 mg/kg) and placed in metabolic cages for 24 hours. (A) accumulative food intake, (B) accumulative locomotion, and (C) accumulative energy balance were assessed. Data are shown as mean ± SEM with n=4 mice per group. Statistical significance was determined by two-way ANOVA followed by Tukey's post hoc test. * $p<0.05$

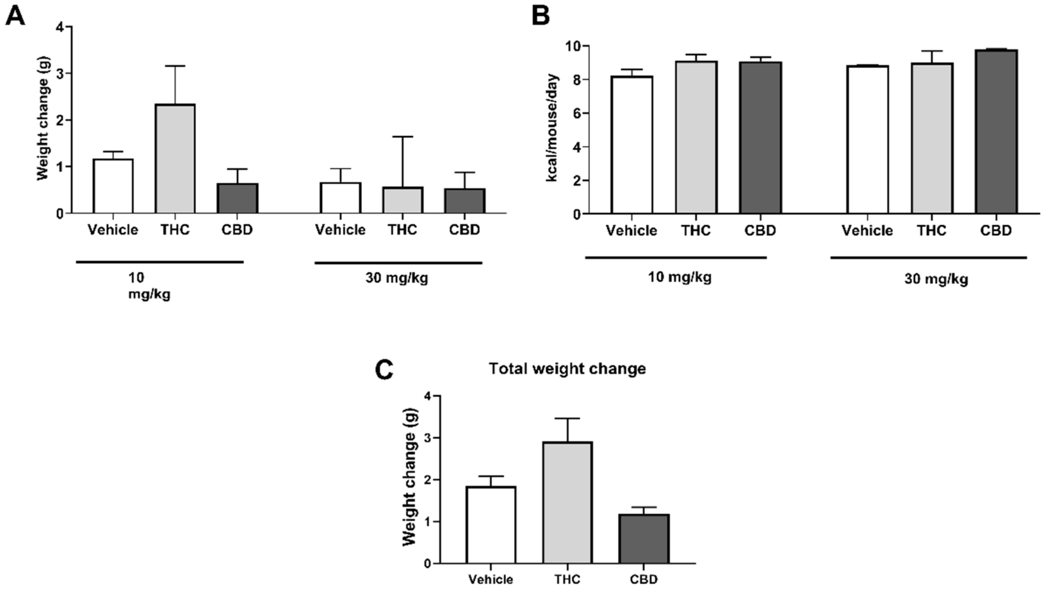


**Figure S2: Effect of THC and CBD on weight gain and caloric intake in mice fed standard diet.** Weight and food consumption were measured twice a week during the curse of the treatment regime. A. Body weight change of mice fed chow diet after 5-week treatment with 10 mg/kg or 30 mg/kg of THC, CBD, or vehicle (n=5), B. Average caloric intake of mice fed a chow diet and treated with 10 mg/kg or 30 mg/kg of THC, CBD, or vehicle (n=5), C. Total weight change of the treatment regime.

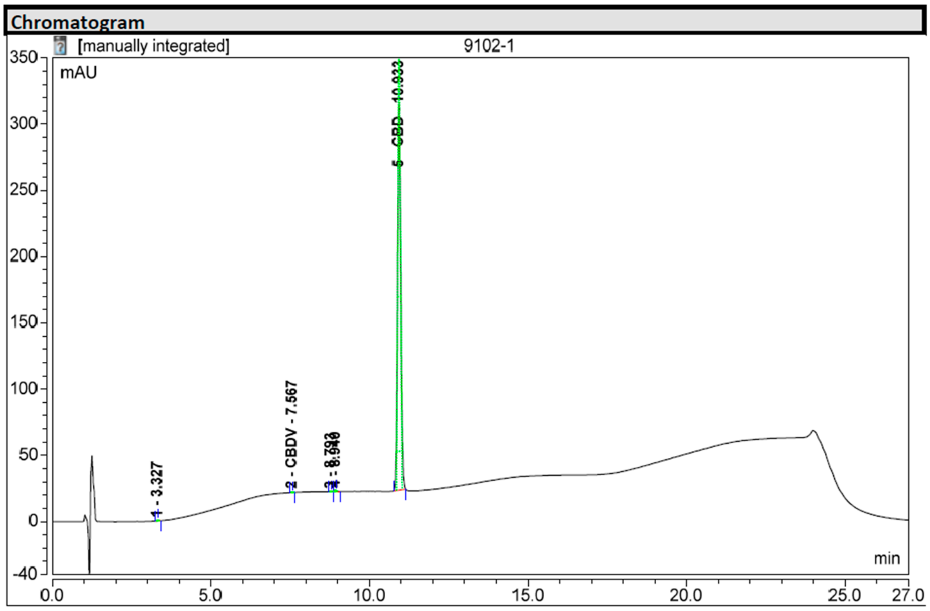


**Peak Results**

| No. | Peak Name | Retention Time<br>min | Area<br>mAU*min | Amount<br>ug/ml | w/w<br>% | Rel. Area<br>% |
|---|---|---|---|---|---|---|
| 1 | | 3.327 | 0.044 | n.a. | n.a. | 0.13 |
| 2 | CBDV | 7.567 | 0.022 | 0.1250 | 0.01 | 0.06 |
| 3 | | 8.793 | 0.004 | n.a. | n.a. | 0.01 |
| 4 | | 8.940 | 0.065 | n.a. | n.a. | 0.19 |
| 5 | CBD | 10.933 | 33.964 | 206.2462 | 14.72 | 99.61 |

**Figure S3: HPLC chromatogram of CBD extract.** Identification of CBD according to analytic standard by LC/HRMS/MS as described in the materials and methods.

## Table S2: Primer list

| Gene | Forward primer / Reverse primer | Accession number |
|---|---|---|
| F4/80 | CCTTTGGCTATGGGCTTCCA | NM_001355722.1 |
| | CCTCAGAACCCACAGTGTCC | |
| TNF-$\alpha$ | GTCTGTGCCTCAGCCTCTTC | NM_013693.3 |
| | GCTTGGTGGTTTGCTACGAC | |
| MCP-1 | GCTGTAGTTTTTGTCACCAAGC | NM_011333 |
| | GTGCTGAAGACCTTAGGGCA | |
| CD14 | CTGAAGCCTTTCTCGGAGCC | NM_009841.4 |
| | GCATAAGCTTCATGGTCGGTAGA | |
| FASN | GTGATAGCCGGTATGTCGGG | NM_007988.3 |
| | TAGAGCCCAGCCTTCCATCT | |
| FADS2 | TTACCAAATGGTCCCAGCGG | NM_019699.2 |
| | GCTTCAAGAACTTGCCCACG | |
| SCD-1 | CCAAGCTGGAGTACGTCTGG | NM_009127.4 |
| | CAGAGCGCTGGTCATGTAGT | |
| CD36 | GCAAAACGACTGCAGGTCAA | NM_001159558.1 |
| | GGCCATCTCTACCATGCCAA | |
| FATP1 | CAGATCGGCGAGTTCTACGG | NM_001357182.1 |
| | TGACCTTGACCAGACGGATG | |
| PPAR-$\alpha$ | CCTCAGGGTACCACTACGGA | NM_001113418.1 |
| | AGCGAATTGCATTGTGTGACAT | |
| ACOX1 | AGTCAAAGGCATCCACCAAAG | NM_001271898.1 |
| | CTATGGGATCAGCCAGAAAGG | |
| CPT1$\alpha$ | GTCTGGCTCTACCATGACGG | NM_013495.2 |
| | CGTGCAAAATAGGTCTGCCG | |
| FATP2 | TGAATGTGTATGGCGTGCCT | NM_011978.2 |
| | AGGTACTCCGCGATGTGTTG | |
| FATP5 | GCCTCTGAGGGACAAACAGG | NM_009512.2 |
| | TCCTACGCGTCGTACATTCG | |
